# "Wrapping myself in cotton wool": Australian women's experience of being diagnosed with vasa praevia

**DOI:** 10.1186/1471-2393-14-318

**Published:** 2014-09-10

**Authors:** Nasrin Javid, Elizabeth A Sullivan, Lesley E Halliday, Greg Duncombe, Caroline SE Homer

**Affiliations:** Faculty of Health, University of Technology Sydney (UTS), Broadway, New South Wales 2007 Australia; School of Public Health & Community Medicine, UNSW Australia, Kensington, New South Wales 2052 Australia; Department of Maternal Fetal Medicine, Royal Brisbane Women’s Hospital, University of Queensland, Herston, Queensland 4006 Australia; Centre for Midwifery, Child and Family Health, Faculty of Health, University of Technology Sydney (UTS), Broadway, New South Wales 2007 Australia

**Keywords:** Vasa previa, Vasa praevia, Ultrasound, Qualitative research, Pregnancy, Obstetrics, Caesarean section

## Abstract

**Background:**

Vasa praevia (VP) is an obstetric condition that is associated with significant perinatal mortality and morbidity. Although the incidence of VP is low, it is one of the few causes of perinatal death that can be potentially prevented through detection and appropriate care. The experience of women diagnosed with or suspected to have VP is largely unknown. The aim of this study was to explore the experiences and impact that a diagnosis or suspected diagnosis of VP had on a group of Australian women.

**Method:**

A qualitative study using a descriptive exploratory design was conducted and Australian women diagnosed with VP were recruited via online methods in 2012. An inductive approach was undertaken and interviews were analysed using the stages of thematic analysis.

**Results:**

Of the 14 women interviewed, 11 were diagnosed with VP during pregnancy with 5 subsequently found not to have VP (non-confirmed diagnosis). Three women were diagnosed during childbirth with one neonatal death. Five major themes were identified: feeling like a ticking time bomb; getting diagnosis right; being taken seriously; coping with inconsistent information; and, just a massive relief when it was all over.

**Conclusions:**

This is the first study to describe women’s experience of being diagnosed with or suspected to have VP. The findings from this research reveal the dilemmas these women face even if their baby is ultimately born healthy. Their need for clear and consistent information, sensitive care, support and continuity is evident. Clinicians can use these findings in developing information, counselling and models of care for these women.

## Background

Vasa praevia (VP) is a condition that carries a high risk of death of an otherwise normal baby. The reported perinatal mortality when the condition is not diagnosed and appropriately managed is approximately 60% [1, 2]. VP occurs when exposed fetal blood vessels, unsupported by the placenta or the umbilical cord, run over the cervix [3]. The exposed fetal vessels are in danger of rupture in late pregnancy or in labour, carrying a high risk of death of the baby. Although the incidence of VP has been estimated to be 1 in 2500 births [3], it is potentially under reported [4, 5]. The incidence of VP in IVF pregnancies is reported to be 1 in 202- 293 births [6, 7]. Although VP is rare, it is a significant pregnancy complication [8, 9]. The risk factors for VP include placenta praevia, low lying placenta [10], multiple pregnancies [11, 12], in-vitro-fertilisation (IVF) [6, 7], bilobed or succenturiate lobed placenta and velamentous insertion of the cord. In recent years, it has become possible to diagnose VP during pregnancy using ultrasound [11, 13–16] with definitive diagnosis being made in the third trimester once the lower segment is formed. Women diagnosed *at risk for* VP during the second trimester need to be confirmed in the third trimester [17] as in some women VP may resolve spontaneously as pregnancy progresses due to an increase in the distance of the fetal vessels from the internal os as the uterus expands. However, there is little international consensus in defining the ‘safe’ distance. A confirmed clinical diagnosis can be made in the third trimester with ultrasonography after lower segment formation, or during labour on visualisation of the fetal vessels in the membranes or on examination of the placenta after birth.

Making the diagnosis and delivering the baby by caesarean section (CS) prior to the onset of labour will prevent the death of the baby in the majority of cases [2, 18]. Despite evidence that VP carries a high perinatal mortality, can be diagnosed prenatally, and that perinatal death can be avoided in most cases by accurate diagnosis and appropriate management, there remains a lack of consensus among medical professionals regarding screening for VP, and on the optimal management of women who are diagnosed with the condition. A number of studies have described approaches to the management of women with VP including hospitalisation at 30-32 weeks and elective CS at 34-35 weeks of gestation [17, 19]. However, there are few national guidelines to support these practices. The Society of Obstetricians and Gynaecologists of Canada (SOGC) developed guidelines in 2009 [20]. The Royal College of Obstetricians and Gynaecologists (RCOG) in the UK updated their “Green Top Guideline 27” for placenta praevia to include guidance about diagnosis and management of VP in 2011 [21]. Currently, there are no national guidelines in Australia although the Royal Australian and New Zealand College of Obstetricians and Gynaecologists (RANZCOG) released a College Statement in 2012, which recommended hospitalisation and elective CS for women diagnosed with VP, but reported *‘no agreed protocols for timing of admission to hospital or timing of elective caesarean section’* [22].

Given the perinatal risks, challenges in the diagnosis of VP and the lack of consensus of optimal management among medical professionals, there is a potential for a diagnosis of VP to lead to significant anxiety and uncertainty among women and has considerable impact on their pregnancies and birth plans, with the potential disruption of their personal lives and those of their families. Despite this, the experience of women diagnosed with or at risk of VP is largely unknown and has not, to our knowledge, been described. The aim of this study was to explore the experiences and impact that a diagnosis or suspected diagnosis of VP had on a group of Australian women.

## Methods

A qualitative study using a descriptive exploratory design was undertaken [23]. Qualitative descriptive studies are particularly useful when seeking to describe people's responses to an event. The method is ideal for research that focuses on the ‘who, what, why, and where’ of events and is useful when investigating previously unexamined experiences. The aim of descriptive exploratory studies is generally to present a comprehensive summary of events as experienced by the participants in the event [23, 24].

Human research ethical approval was received from the University of New South Wales Human Research Ethics Committee prior to commencement.

### Setting and participants

Women were recruited during 2012 through the International Vasa Previa Foundation (IVPF). The IVPF is a not-for-profit organisation providing information and support about VP and education for consumers and clinicians. A notice was put on the Facebook page of the IVPF and an email was sent to members of the closed ‘Vasa praevia Yahoo Group’ in July 2012; asking interested women who were diagnosed with this condition and had their care in Australia to contact a researcher.

To be eligible, a woman had to have been diagnosed with VP during pregnancy or childbirth while living in Australia, speak English and be willing and able to provide consent.

### Data collection

Data were collected using one-to-one interviews which were conducted by telephone. Prior to the interview, women were sent study information leaflets and asked to provide written consent to participate. Interviews were audio-recorded. The interviews took between 30-90 minutes with a mean of 50 minutes and were guided by a series of semi-structured open-ended questions. One author, LH, an experienced qualitative researcher, undertook all the interviews. Fifteen women volunteered for the study and were interviewed. One woman was excluded from the analysis as she had her diagnosis of VP and pregnancy care in another country. A total of 14 women were included in the analysis.

**Trigger questions used in the interviews**

- Can you talk about your experience?
- How were you diagnosed?
- How did you and your partner react to the news?
- What was it like to receive a diagnosis?
- How were you cared for and what did you think about this care?
- What did you previously know about vasa praevia?
- Can you talk about your birth and your baby?
- When you think back, is there anything that you think was handled well and anything that should have been done differently?

### Data analysis

Interviews were transcribed verbatim. An inductive approach was taken and data was analysed using the stages of thematic analysis [25]. Audio interviews were listened to and transcripts were read through several times by two of the authors. Two researchers independently coded the transcripts. The researchers met on a regular basis to discuss the concepts and developed these into codes and themes. Women’s words have been chosen to describe the themes.

Verification of the final codes and themes was undertaken by inviting a consumer who had experienced vasa praevia and had many years of experience in talking with women in this situation to read the analysis for content and face validity.

## Findings

Women were aged from 27-38 years at the time of diagnosis, had a singleton pregnancies, and all were married or in a long term relationship with a male partner. Eleven women were diagnosed with VP during pregnancy with five not confirmed to have VP on subsequent follow up ultrasound. One woman who was diagnosed during pregnancy did not have confirmation of VP in late pregnancy; however VP was confirmed at birth.

Three women were diagnosed at the time of childbirth. One of these had an elective CS due to a previous CS; and the other two had vaginal bleeding after an induction of labour and had emergency CSs. In this latter group, there was one neonatal death within 48 hours of birth and one baby who had an Apgar score of 1, 3 and 5 at one, five and ten minutes of birth, but was successfully resuscitated. Table 1 provides details regarding the women’s pregnancy characteristics including the gestation at diagnosis, presence of risk factors for VP and birth details. All except two women had at least one risk factor for VP, as described in the literature [3, 4, 7, 8, 10–12].

**Table 1:** **Pregnancy and diagnosis details of the women (n=14) by three groups**

Group	Gestational age range risk of VP first identified or diagnosed (weeks)*	Gravidity & parity ^#^	Risk factors	Mode of birth	Gestation at birth	Time between interview and birth (months)
1. Antenatal diagnosis: confirmed during pregnancy	U/S (18-20)	G2P1	IVF, Placenta praevia	Elective CS	35	< 6
	U/S (32-34)	G1P0	IVF, Placenta praevia	Elective CS	36	> 12
	U/S (32-34)	G2P1	Bi-lobed, Low lying placenta	Elective CS	37	< 6
	U/S (18-20)	G1P0	IVF, Placenta praevia	Elective CS	35+4	> 12
	U/S (26-28)	G1P0	Placenta praevia	Urgent CS	35+4	< 6
	U/S (18-20)	G1P0	Velamentous cord insertion	Elective CS	37	< 6
2. Antenatal diagnosis: not confirmed at follow up ultrasound(s)	U/S (26-28)	G1P0	Tri-lobed placenta, low lying placenta	Elective CS	37+5	Unknown
	U/S (18-20)	G2P1	Placenta praevia	Elective CS	37	<6
	U/S (18-20)	G1P0	low lying placenta	NVB	40	< 6
	U/S (32-34)	G2P1	low lying placenta	Elective CS	37 +4	> 12
	U/S (18-20)	G1P0	Velamentous cord insertion	Emergency CS	36	> 12
3. Diagnosed during birth	CS	G4P1	None	Elective CS	37 +4	> 12
	Vaginal bleeding and CS	G1P0	IVF	Emergency CS	40+2	< 6
	Vaginal bleeding and CS	G1P0	None	Emergency CS	41	> 12

*Gestational age range is presented to protect women’s identity.

#Gravidity/parity at the pregnancy VP was diagnosed.

US: Ultrasound, G: Gravidity P: Parity, CS: Caesarean Section, IVF: In-vitro-fertilisation.

Six major themes were identified from the data. These were: (1) *Feeling like a ticking time bomb*; (2) *Getting the diagnosis right*; (3) *Being taken seriously*; (4) *Coping with inconsistent information*; (5) *Just a massive relief when it was all over*; and, (6) *Making the experience better*. Quotes from women are identified in quotation marks and *italics* to highlight the themes. Women are identified by one of the three groups in which they fell: (1) antenatal diagnosis; confirmed; (2) antenatal diagnosis; not confirmed in the follow up ultrasound; (3) diagnosed at the time of the childbirth. For example, participant 1-4 represents group one, fourth interviewee.

## Results

### ‘Feeling like a ticking time-bomb’

*“After finding out, it was hard to be relaxed and you felt like a ticking time-bomb – that you weren’t sure what you could do” (participant 2-4)*

*‘Feeling like a ticking time-bomb’* expressed the fear, anxiety, uncertainty and distress that women felt, particularly those diagnosed in pregnancy regardless of their final diagnosis. Most women expressed fear when they were diagnosed, describing it as ‘*overwhelming and scary’* and ‘*worrying’*. They frequently used words such as ‘*shocked’* , ‘*traumatised’* and a number were *‘unable to think positively about the pregnancy or the baby’*. One woman said:

*“I did nothing, went nowhere… I just stayed at home. I didn't go back to work… I pretty much wrapped myself in cotton wool and stayed at home”. (participant 1-4)*

Fear of losing the baby further traumatised the pregnancy for most women. Some expressed that they could not relax and enjoy their pregnancy, describing their experience as *‘just kind of life changing because you are so close to death and living with that’*. One woman reported:

*“I became very cautious about any physical exertion; didn’t ever go very far from home (lived 10 mins from the hospital) and furthest I walked for the rest of my pregnancy was 0.25-0.5 km up the road. I am usually a very positive and optimistic person, but I found it quite hard to be positive especially in the early weeks after diagnosis”. (participant 2-1)*

Another woman commented:

*“I completely gave up exercise … I was constantly thinking about the baby. My behaviour did change after the diagnosis. I didn't want to do too much strenuous exercise in case I ruptured something”. (participant 1-6)*

Some women could not connect with the baby and were afraid of bonding as they felt they might lose the baby, as this women expressed:

*“I didn't feel excited about the pregnancy. I couldn't pick a name. I didn't buy anything. I didn't set up a room. I just didn't want to really do anything because I was frightened of the prospects that the baby wouldn't make it”. (participant 1-1)*

Stress was strongly expressed by almost all women diagnosed during pregnancy. One described the cumulative impact of the stress as the pregnancy progressed, saying:

*“… there was constant stress and worry … this was just building up to the worst possible day [the birth]. I was just so terrified of losing the baby or having a baby that had a brain injury”. (participant 1-1)*

The three women who were diagnosed at the time of birth also experienced shock and trauma, although this occurred later than those diagnosed in pregnancy. One woman, despite not experiencing fetal bleeding due to having an elective CS, and giving birth to a healthy baby, still relayed that she ‘*spent so much time in shock*’ and was ‘*horrified’*, after the diagnoses was made. She felt she came close to losing her baby and could not stop thinking about ‘*what could have happened’*.

### ‘Getting the diagnosis right’

*“Vasa praevia is not an accident. It is not something where you just roll the dice and take your chances and it is something that can be diagnosed”. (participant 3-1)*

Many women, even those with a changed diagnosis, were very grateful that the initial diagnosis of VP was made. Statements like: ‘*I felt lucky to have the antenatal diagnosis*’, and, ‘*I was grateful to have the diagnosis and a plan to stay in hospital*’ expressed the relief at having a diagnosis and a plan of care. One woman talked about the sonographer who did the first diagnosis of VP and ‘*thought he was quite a hero for finding it’*. She went on to say:

*“I was pleased with the sonographer picking it up. We thought it was amazing and impressed and we were really glad and honoured to have him pick it up, the initial diagnosis”. (participant 2-5)*

In contrast, some women questioned the health system for not picking up the VP during pregnancy. One woman who was diagnosed during a CS and her baby was born healthy, considered herself *‘lucky’*, but said:

*“It just really made me, not angry, but I just didn't understand how it could be missed, like how it could not be important to find out. I said to the obstetrician that I want to know how this got missed. Why isn't this looked for? No-one wants to look at the placenta and the umbilical cord and how they insert in to each other and match up. You know, that's ridiculous. Especially when you are paying for extra hospital cover and private health insurance and all that stuff and to still have someone say well it is an economic decision whether your baby gets a chance of being born alive or not”. (participant 3-1)*

Even when the diagnosis was made, there was inconsistency in the management. All but one woman had follow-up ultrasounds to confirm the diagnosis at later gestation. The exception explained:

*“…at the 18 week scan that [VP] was confirmed. There was some discussion that I might have a 28 week scan but I didn't have that because my doctor was confident that nothing would have changed or that the management would be any different…”. (participant 1-1)*

Women were very keen for their caregivers to ‘get the diagnosis right’. This was particularly challenging for the five women whose diagnosis was changed. These women felt ‘*confused’* as they perceived a lack of confidence within the clinicians. One woman felt ‘*extraordinarily upset’* about the changed diagnosis and did not trust that it was not VP. She did not feel safe to have a natural birth and questioned *‘the distance that makes it not vasa praevia and when does it become velamentous cord insertion’.* She explained:

*“I wanted him [first sonographer] to be involved again and to double check and felt that there wasn't a consensus absolutely with the doctors about it… they didn't seem to kind of agree on the way in which it [the distance of fetal vessels from internal os] was being measured. So we opted for a caesarean and continued to be monitored. I found those weeks almost the most stressful”. (participant 2-5)*

Similarly, another woman commented:

*“The obstetrician said that it's not really vasa praevia anymore and that I could go home if I wanted. So we were really confused then. He couldn't tell me, when we said “well what is a safe distance?” he couldn't say. Well it's now 3 cm away …, again there didn't seem to be anything to guide us on that and no-one really wanted to commit to saying anything.” (participant 2-2)*

One woman who was diagnosed at 32 weeks and then had the diagnosis changed at 36 weeks, felt ‘*confused’* as she perceived her obstetrician was not confident with this saying:

*“the baby was really far down. So it was hard to do the ultrasound. … They [sonographers] saw that there was something there, but they didn’t think it was vasa praevia.” (participant 2-4)*

She was advised to have another ultrasound at 38 weeks but chose to have an elective CS and VP was confirmed at birth. She reflected on this experience:

*“They said we can do another ultrasound in two weeks [at 38 weeks] and if we still don't find anything we think you should have a natural birth…. The obstetrician obviously noticed that I was nervous about it and so on but I don't feel like they gave me enough support or information about it. He told me that he knew what it was and he even lost a baby to it once. So I was surprised that he didn't take it more seriously than he did… I was quite disappointed with the lack of information or advice” (participant 2-4)*

### ‘Being taken seriously’

*“They [my carers] were absolutely fantastic. It was very uncertain, the last 20 weeks of pregnancy, but they kept a good eye on me and I had scans to check the location of the blood vessels …. So I did feel very well cared for and felt like it was being taken very seriously which was great”. (participant 1-6)*

Some women felt they were *‘taken seriously’* and were very happy with their care. One woman who received enough information about VP and was given a plan, ‘*felt confident’* with her doctors. She expressed that she ‘*had good care and good management of the vasa praevia’* , had ‘*a lovely birth’* and considered herself ‘*very lucky’*. Another felt *‘incredibly fortunate, supported and confident’* with her doctors. She reported that ‘people *were taking steps to ensure that my baby was born safely’.* Another woman who was hospitalized at 34 weeks expressed her satisfaction with her admission and care saying:

*“They were really good at answering questions… They delivered the baby safely and kept me safe and the baby safe for the last few weeks… they were just incredible” (participant 1-3)*

However, many more felt that they were *‘not taken seriously’* as they were dissatisfied with the way the diagnosis was disclosed or explained to them. One woman perceived *‘just given the initial news, being delivered in the way it was, it kind of set up a bit of trauma for the rest of it’.* She further explained her response saying:

*“He sort of rattled off it meant and went on to say how he had delivered quite a lot of dead babies with this condition. That was really not what you need to hear. It was probably just the way he explained it. He said “if you were in the bush in Africa you wouldn't have a live baby” kind of thing. I found it was a little bit insensitive for him to say something like that. I think he was just trying to make light of the situation but I didn't find that all that helpful”. (participant 1-6)*

Most women felt that they did not receive enough information and advice at the time of diagnosis from their health care providers and did not understand the risks. Statements like ‘*I just wanted to know’ , ‘I always feel better about things, when I know all the ins and outs of it’ and ‘I wanted the facts’* expressed women’s need for information. For example:

*“I didn't think he [the obstetrician] was treating it seriously enough. He sort of said “see this as a hiccup, don't see this as a catastrophe” which is great. But it didn't really help when you don't quite understand exactly what the problem is”. (participant 1-6)*

Almost all the women had no prior knowledge of VP. This ‘*lack of prior knowledge made it confusing*’ and most knew no-one who had the condition before with one woman being certain that ‘*no one else has experienced this before*’. Women found it ‘*hard to explain to others’* and one woman said that ‘*none of my friends knew about VP’*. Even when women were given information, some could not understand what they were told, for example:

*“I think that my doctor probably takes for granted some basic medical knowledge. Whereas I don't have any … I needed somebody to sit down and explain it to me very basically”. (participant 1-1)*

Some felt that their doctors were ‘passionate’ and ‘*had good bedside manners’* , but *‘were not informed’* about VP and were unable to convey the seriousness of the situation. One woman who had IVF pregnancy commented that ‘*there was no plan*’ and felt she was giving information to her doctor:

*I would have much preferred if the doctor told me what the go was, not me handing information to him and saying you should read this so you can save my baby's life. Then [for] him [to] say to me ‘well when do you want to have the baby?’… Well I don't know, I'm not the doctor. I'm just this person who has tried my heart out to have a baby and I'm finally pregnant”. (participant 1-4)*

Most women talked about midwives being ‘*fantastic’* but at the same time felt that most were uninformed about VP and again did not realise the serious nature of the condition. A number of women were uncertain whether the midwives ‘*understood the difference between VP and placenta praevia*’ and therefore would mistake any vaginal bleeding for a placenta praevia and would not take immediate action. This made a few women feel that they would *‘not be taken seriously if they bled’*:

*“I was in a room of women who had low lying placentas and placenta praevia. My biggest worry was that if I rang the buzzer and said I'm having a bleed, that I would not be treated correctly, because not every midwife knew what was going on with me…” (participant 1-2)*

### ‘Coping with inconsistent information’

*“It would make everything so much better if we could have someone tell you what was going on. These are the rules, this is what happens, this is what we do … Whereas everything was all, “oh we'll just take it day by day”. (participant 1-4)*

Women continually reported receiving inconsistent information about VP, their own diagnosis and the best management plan. There were also inconsistency in information including the timing of ultrasounds; safe distance to be away from the hospital; need for, and timing of hospitalisation; fetal monitoring; and, timing of the birth. Timing of birth was the area of most inconsistency and confusion with more than 20 direct comments about this issue. There were differences between clinicians within the same hospital, between hospitals, with timing changed for individual women during pregnancy. One woman clearly articulated her concerns:

*“They said that I will have a CS at 39 weeks. My obstetrician said “we can’t do it early”. She told me that it was their procedure. I just don’t know whether it is public hospital procedure or Australian procedure. The first doctor told me it would be at 37 weeks and then this one told me it would be 39 weeks…then I don’t understand why they’ve given me inconsistent information.” (participant 2-3)*

Two women who had an elective CS planned for 38.5 weeks, talked about their concerns and worries of having a late CS as they had found that the recommended time for birth was 35-36 weeks on the internet. One perceived that the doctor handed over the decision making for timing of birth, when she chose to have CS at 35.5 weeks. She explained:

*“He said ‘I'm leaving the decision in your hands, when do you want to do the CS?’… I felt then that if there was going to be something wrong with the baby it was going to be all my fault because I was the one who said “I want my CS done on this day”. (participant 1-4)*

Another woman spent a sleepless night before her elective CS researching her decision on the internet as she was concerned that the timing was not right:

*“My doctor had, all through the pregnancy, discussed going for 36 weeks but then because of my degree of anxiety about it all, he said that he did further research and decided he wanted to do it at 35 weeks. I sort of switched back then and had a lot of reservations about doing that because I was worried about the effect of the baby being born that week earlier. I was very upset in the end of having the baby at 35 weeks. I think my doctor wanted the birth over and done with and for somebody else to take care of the baby”. (participant 1-1)*

The internet also provided inconsistent information about timing of the birth. For some women, the information on the internet was different to what their doctors provided. This inconsistency resulted in lack of confidence and trust regarding their care and in the decision making. One woman commented that “*it's hard because you don't know who to trust*”. Another woman said that *“because of the inconsistency, it is making me even more worried.”*

In contrast, those women who received consistent information felt confident with one saying:

*“I was quite confident in the way the doctors handled things. They were quite reassuring and booked me in at 36 weeks to have a caesarean. I did my own bit of research with the International Vasa Praevia Foundation. Their recommendation was 35 weeks not 36. So I did question that with my doctors and I was quite reassured that the extra week was OK if I was in hospital and if nothing had happened”. (participant 1-2)*

Women whose diagnosis was changed struggled even more with the information and advice and mostly did not trust their new diagnosis. Some woman in this group despite being told that they did not have VP, didn’t get enough reassurance and advice which added to their level of uncertainty. Typical comments were: ‘*they didn’t tell me why I shouldn’t worry about it’* and ‘*I didn’t feel they were consistent’*. One woman reported:

*“The obstetrician said it’s not vasa praevia anymore and that I could go home if I wanted. So we were very confused. He couldn’t tell me … “well what is a safe distance?” he couldn’t say…” (participant 2-2)*

She consulted a VP group in the UK and an obstetrician in another part of Australia and neither could say ‘*what a safe distance is’ ,* so she decided to stay in hospital saying:

*there didn't seem to be anything to guide us on that and no-one really wanted to commit to saying anything. So we don't know if we did the right thing or not. Of course I didn't want to stay in hospital and that actually made it worse because I had a daughter at home. I knew they needed me at home. I was really torn whether or not I was doing the right thing and then to be made to feel that I was possibly taking up a bed and that I didn't really need to be there and I didn't really have vasa praevia, I was completely confused as to where I should be”. (participant 2-2)*

Women used the internet for information about vasa praevia including the need for admission to hospital and timing of birth. Women were either ‘*encouraged to read online’* or ‘*advised to not go home and google’* by their doctors. However, all women in this study used internet to get information regarding VP. One woman said that her doctor encouraged her to read on the internet but then disregarded the information and advice she found, for example:

*“When he saw that I was quite worked up about it, he was like “yeah, yeah, but don't believe everything you read on the internet”. (participant 2-4)*

Several women said that the stories on the internet were ‘*horrible’*, ‘*frightening’*, a ‘*nightmare’* and about *‘people who have lost babies’*. But for at least five women, having knowledge helped them process the experience and reduced their fears. Women felt like: ‘*knowledge reduced my fear’*; and ‘*I was not in in shock due to having the knowledge’*; and, ‘*I was less distressed because I knew about it’* and ‘*not too shocked really because I knew it was a possibility*’. Some women said that reading those stories on internet ‘*helped me to know how lucky I am*’; and ‘*helped me understand and know about different outcomes’*.

### ‘Just a massive relief’ when it was all over

*“It was just a massive relief… he was breathing fine … we had this healthy little boy”. (participant 1-2)*

Ultimately relief came once the baby was safely born. Nine of the 14 women expressed relief at hearing the baby cry for the first time. Women made comments like: ‘*she was out and that was all my concern. She was really healthy’*; ‘*I could feel the tension melt away after she was born safely*’; ‘*once she was out I was relieved - phew, it was all over*’ and ‘ *once she was out I was phew … she's not going to die now, she'll be fine*’. Another said:

*“I was just so relieved, all the tension and knowing that everything went well and you could finally relax and enjoy the baby.” (participant 2-4)*

One woman clearly expressed this relief as:

*“We were so incredibly in raptures in having her safe that definitely the first six months it was just like utopia. It was just so beautiful … we got her here. So that joy bubble lasted a really long time. Almost like an after effect that was also relaxing. Such euphoria in having her safe”. (participant 2-5)*

Some women felt relief during pregnancy when they had a plan in place or when they were in hospital. One woman ‘*felt really safe’* during her admission in hospital and considered herself *‘fortunate and lucky’*. For example:

*“Once I was there in hospital I didn't really have a concern that anything was going to go pear-shaped in terms of losing the baby”. (participant 1-3)*

‘Just a massive relief’ when it was all over, describes that most women were ‘*desperate to get there and to know that everything is going to be OK’*. One woman who was uncertain about her changed diagnosis described her relief when she was told she would have an emergency CS:

*“When they said to us “it's going to come” we thought “thank God it's going to be over, just get her out and get her safe”. (participant 2-5)*

This was in contrast to the woman who was diagnosed during childbirth. She had ruptured membranes, vaginal bleeding and emergency CS, but her baby died. She described the time when she saw her baby for the first time after being woken up from CS:

*“They were ventilating him by hand. He looked pink, so I couldn't believe that there was actually anything really – I just couldn't reconcile in my mind that there was something wrong… Then they told me that they [doctors] had to revive him. They gave him two blood transfusions and he suffered a couple of heart attacks on the table but that he was basically brain dead and that there was little more that they could do for him”.(participant 3-3)*

### ‘Making the experience better’

The women in our study were reflective about their experiences and thoughtful about what they would have wanted for their care and what women in the future would want. Many of these reflections included the issues already discussed in the five themes above. Additional issues included the importance of having a plan; the need for support and someone to talk to and the value of continuity of caregivers.

Many women felt isolated in their experience with few of their friends understanding their experiences or even the diagnosis. They would have valued someone to talk to who understood what they were going through. Most women commented that they preferred to talk to somebody who was informed about VP. However, this was not offered through the health system as one woman commented:

*“And at the hospital staff was really good. But I don't know if they even knew about it. If they did, they didn't say anything. But they were very nice at the hospital. But yeah, I guess lack of information and even being told if there was someone you could call or talk to. That never even got offered. I think I found that pretty hard”. (participant 2-4)*

Continuity of care was also valued by women. One woman said:

*“… the one-on-one care. For me that was huge, being able to have that constant one person who knew what was going on ……… someone that I trusted because I had had her throughout my whole pregnancy… ” (participant 1-2)*

## Discussion

This is the first study undertaken to explore the experiences of women with VP, either diagnosed during pregnancy or in childbirth. Our study included a spectrum of women – some were diagnosed in pregnancy, others did not have this confirmed as the pregnancy progressed with a minority of women diagnosed in childbirth, including one woman who experienced a neonatal death. The main findings of the study were the lack of information and certainty for women and the fear and anxiety that this created.

Receiving consistent information is a challenge for women with VP given the lack of guidelines and evidence. There is still considerable uncertainty about the optimal care of these women [26]. In many centres in Australia there is no agreed protocol for screening, diagnosis or even management once VP is confirmed and, anecdotally, there seems to be limited consistency in approach between obstetric specialists and/or centres. Lack of knowledge and need for increasing awareness about diagnosis and optimal management of VP have been previously reported [27–29]. A survey of 128 obstetricians in UK in 2006 showed that 59% of the obstetricians would not offer hospitalisation and although 80% offered elective CS for women diagnosed with VP, 46% would not perform a CS until completed 38 weeks gestation [27].

Increasingly, consumers are accessing the internet for health-related information. Several studies reveal that people are using information from the internet to make decisions regarding their health [30, 31]. The internet as a source of health information has been reported *to “empower patients and increase their sense of control over their disease”* [32], but might also alter the clinician-patient relationship. Our study shows that some women used the internet as a source of current information for VP and had uncertainty in who to trust: their care providers or the internet in relation to the best management [33]. Some of the women in our study perceived that they were more informed about their particular condition (VP) than their care providers. This struggle for the ‘right’ information sets up difficult dynamics for relationship between the women and their clinicians.

The women in this study for whom the diagnosis was not confirmed on a later ultrasound expressed additional worries. In 2014, Rebrarber et al. reported the incidence of VP was 1 in 1000 pregnancies. In this study authors concluded that 24% of women diagnosed with VP during the second trimester did not have confirmation of VP across gestation. However, all women diagnosed in the third trimester had confirmed diagnosis [18]. In our study, women who were not confirmed to have VP still reported considerable stress especially as there seemed to be a lack of certainty and evidence about the “safe” distance of the exposed vessels to the os. One woman in our study who was diagnosed at 32 weeks had another ultrasound at 36 weeks that did not confirm VP. However, VP was diagnosed at elective CS. It has been reported in the literature that in late pregnancy there is a possibility of poor visualisation of fetal vessels in the ultrasound that may prevent a confirmation of VP [14, 22].

Other studies have shown how diagnosis ambiguity in general creates stress. For example, a prospective study of women who had prenatal diagnosis of fetal structural anomaly by ultrasound reported that ambiguity regarding diagnosis [34], as well as uncertainty about prenatal outcome of the fetus to be associated with high psychological distress [35]. Another study showed that false positive soft markers in prenatal ultrasound screening provoked maternal anxiety during pregnancy [36] and reduced early mother-infant interaction at 2 months after giving birth, although the infant was normal.

Many women in our study expressed fear, stress and anxiety during their pregnancy and also felt lack of control in terms of decision-making. Several studies have focused on anxiety in pregnancy and have reported antenatal anxiety [37–39] and worry [40] to be important predictors of postnatal depression. For example, Lee at el. 2007 [37] reported increased association between antenatal anxiety and postnatal depression with the progression of pregnancy. It has also been shown that there is strong association between postnatal depression and parenting stress [41].

It is possible that having considerable fear and anxiety has effects on the pregnancy and the new mothering experience for the women. Some studies found that antenatal anxiety had negative effects on maternal-infant attachment. Antenatal anxiety has also been shown to be associated with difficult infant temperament [42] and emotional and behavioural difficulties in childhood [43–45]. This is a significant issue for the long-term health of both mother and baby and needs to be addressed in future research and practice models.

The findings of this study demonstrate the need for increased awareness of VP within sonographers, doctors and midwives. A careful discussion is encouraged between the woman and her obstetrician so that the woman understands her diagnosed condition, and her management plan. Dissemination of current available information and establishment of the national guidelines regarding accurate diagnosis and efficient management will help to achieve optimal care.

We present a small qualitative study of a very rare condition and the findings are presented from the perspective of these women. We did adhere to the guidelines for Qualitative research review guidelines (RATS) which includes relevance of study question; appropriateness of qualitative method; transparency of procedures; and, soundness of interpretive approach [46]. As with all qualitative studies based on individual recollections, there is the potential for recall bias. There is also possibility of selection bias as women who volunteered to be interviewed might be different to those who did not although this is a recognised limitation of qualitative studies, especially those of rare conditions. We recruited women through an online foundation which may mean that the findings are not generalizable to other women with VP. However, internet access and usage in Australia is high and many people (62%) use the internet for health information and support [47]. There is a need for larger study to confirm these findings. We did not interview the clinicians involved in the care of these women as the purpose of this study was to investigate women’s experience of being diagnosed with vasa praevia. However, their perceptions will be important to explore in future research. In particular, the importance of consistent information needs to be further explored with clinicians.

## Conclusion

Our study has shown that women with a diagnosis of VP, even if it is subsequently not confirmed, have considerable fears and anxieties related to their pregnancy and birth. Women also found the lack of clear information regarding VP added to their distress. Provision of clear evidence-based information and sensitive support to women soon after the initial diagnosis and throughout the pregnancy is indicated to assist women and their families coping with this serious but rare condition.

Further research needs to address the gaps in knowledge regarding the diagnosis and management for women with VP. The development of national or international guidelines may assist clinicians to provide high quality care for women with this significant pregnancy complication.

## Abbreviations

CS: caesarean section
G: gravidity
IVPF: International Vasa Previa Foundation
IVF: in-vitro-fertilisation
P: parity
VP: Vasa praevia
US: Ultrasound.

## References

[1] Fung TY, Lau TK (1998). Poor perinatal outcome associated with vasa previa: is it preventable? A report of three cases and review of the literature. Ultrasound Obstet Gynecol.

[2] Oyelese Y, Chavez MR, Yeo L, Giannina G, Kontopoulos EV, Smulian JC, Scorza WE (2004). Three-dimensional sonographic diagnosis of vasa previa. Ultrasound Obstet Gynecol.

[3] Oyelese KO, Turner M, Lees C, Campbell S (1999). Vasa previa: an avoidable obstetric tragedy. Obstet Gynecol Surv.

[4] Hasegawa J, Nakamura M, Ichizuka K, Matsuoka R, Sekizawa A, Okai T (2012). Vasa previa is not infrequent. J Matern Fetal Neonatal Med.

[5] Donnolley N, Halliday LE, Oyelese Y (2013). Vasa Praevia: a descriptive review of existing literature and the evolving role of ultrasound in prenatal screening. AJUM.

[6] Baulies S, Maiz N, Munoz A, Torrents M, Echevarria M, Serra B (2007). Prenatal ultrasound diagnosis of vasa praevia and analysis of risk factors. Prenat Diagn.

[7] Schachter M, Tovbin Y, Arieli S, Friedler S, Ron-El R, Sherman D (2002). In vitro fertilization is a risk factor for vasa previa. Fertil Steril.

[8] Cipriano LE, Barth WH, Zaric GS (2010). The cost-effectiveness of targeted or universal screening for vasa praevia at 18-20 weeks of gestation in Ontario. BJOG.

[9] Gardosi J, Kady SM, McGeown P, Francis A, Tonks A (2005). Classification of stillbirth by relevant condition at death (ReCoDe): population based cohort study. BMJ.

[10] Francois K, Mayer S, Harris C, Perlow JH (2003). Association of vasa previa at delivery with a history of second-trimester placenta previa. J Reprod Med.

[11] Derbala Y, Grochal F, Jeanty P (2007). Vasa previa. JPrenatal med.

[12] Gandhi M, Cleary-Goldman J, Ferrara L, Ciorica D, Saltzman D, Rebarber A (2008). The association between vasa previa, multiple gestations, and assisted reproductive technology. Am J Perinatol.

[13] Catanzarite V, Maida C, Thomas W, Mendoza A, Stanco L, Piacquadio KM (2001). Prenatal sonographic diagnosis of vasa previa: ultrasound findings and obstetric outcome in ten cases. Ultrasound Obstet Gynecol.

[14] Daly-Jones E, John A, Leahy A, Mckenna C, Sepulveda W (2008). Vasa Praevia; a Preventable Tragedy. Ultrasound.

[15] Lee W, Kirk JS, Comstock CH, Romero R (2000). Vasa previa: prenatal detection by three-dimensional ultrasonography. Ultrasound Obstet Gynecol.

[16] Marr S, Ashton L, Stemm A, Cincotta R, Chua J, Duncombe G (2013). Vasa praevia: ultrasound diagnosis at the mid-trimester scan. AJUM.

[17] Oyelese Y, Smulian JC (2006). Placenta previa, placenta accreta, and vasa previa. Obstet Gynecol.

[18] Rebarber A, Dolin C, Fox NS, Klauser CK, Saltzman DH, Roman AS (2014). Natural history of vasa previa across gestation using a screening protocol. J Ultrasound Med.

[19] Robinson BK, Grobman WA (2011). Effectiveness of timing strategies for delivery of individuals with vasa previa. Obstet Gynecol.

[20] Gagnon R, Morin L, Bly S, Butt K, Cargill YM, Denis N, Hietala-Coyle MA, Lim KI, Ouellet A, Raciot MH, Salem S, Hudon L, Basso M, Bos H, Delisle MF, Farine D, Grabowska K, Menticoglou S, Mundle W, Murphy-Kaulbeck L, Pressey T, Roggensack A, Diagnostic Imaging Committee (2009). Guidelines for the management of vasa previa. J Obstet Gynaecol Can.

[21] Royal College of Obstetricians and Gynaecologists (2011). Placenta Praevia, Placenta Praevia Accreta and Vasa Praevia: Diagnosis and Management. Royal College of Obstetricians and Gynaecologists. Placenta praevia, placenta praevia accreta and vasa praevia: diagnosis and management.

[22] *Royal Australian and New Zealand College of Obstetricians and Gynaecologists. College Statement C-Obs 47 Vasa praevia*. Melbourne: RANZCOG; 2012.

[23] Sandelowski M (2000). Whatever happened to qualitative description?. Res Nurs Health.

[24] Sandelowski M (2010). What's in a name? Qualitative description revisited. Res Nurs Health.

[25] Saldaña J (2012). The coding manual for qualitative researchers.

[26] Wood P (2013). Vasa praevia and placenta praevia screening in pregnancy. UK National Screening Committee.

[27] Ioannou C, Wayne C (2010). Diagnosis and management of vasa previa: a questionnaire survey. Ultrasound Obstet Gynecol.

[28] UK National Screening Committee (2013). Screening for Vasa Praevia and Placenta Praevia.

[29] Nishtar A, Wood PL (2012). Is it time to actively look for vasa praevia?. J Obstet Gynaecol.

[30] Brotherton JML, Clarke SJ, Quine S (2002). Use of the Internet by oncology patients: its effect on the doctor-patient relationship. Med J Australia.

[31] Pew I, American Life P, Fox S, Rainie H: *Vital decisions : how Internet users decide what information to trust when they or their loved ones are sick : plus a guide from the Medical Library Association about smart health-search strategies and good web sites*. Washington, D.C: Pew Internet & American Life Project;

[32] Broom A (2005). Virtually he@lthy: the impact of internet use on disease experience and the doctor-patient relationship. Qual Health Res.

[33] Anderson JG, Rainey MR, Eysenbach G (2003). The impact of CyberHealthcare on the physician-patient relationship. J Med Syst.

[34] Kaasen A, Helbig A, Malt UF, Naes T, Skari H, Haugen G (2010). Acute maternal social dysfunction, health perception and psychological distress after ultrasonographic detection of a fetal structural anomaly. BJOG.

[35] Aite L, Zaccara A, Trucchi A, Brizzi C, Nahom A, Iacobelli B, Capolupo I, Bagolan P (2009). When uncertainty generates more anxiety than severity: the prenatal experience with cystic adenomatoid malformation of the lung. J Perinat Med.

[36] Viaux-Savelon S, Dommergues M, Rosenblum O, Bodeau N, Aidane E, Philippon O, Mazet P, Vibert-Guigue C, Vauthier-Brouzes D, Feldman R, Cohen D (2012). Prenatal ultrasound screening: false positive soft markers may alter maternal representations and mother-infant interaction. PLoS One.

[37] Lee AM, Lam SK, Sze Mun Lau SM, Chong CS, Chui HW, Fong DY (2007). Prevalence, course, and risk factors for antenatal anxiety and depression. Obstetrics & Gynecology.

[38] Robertson E, Grace S, Wallington T, Stewart DE (2004). Antenatal risk factors for postpartum depression: a synthesis of recent literature. Gen Hosp Psychiatry.

[39] Coelho HF, Murray L, Royal-Lawson M, Cooper PJ (2011). Antenatal anxiety disorder as a predictor of postnatal depression: a longitudinal study. J Affect Disord.

[40] Austin MP, Tully L, Parker G (2007). Examining the relationship between antenatal anxiety and postnatal depression. J Affect Disord.

[41] Leigh B, Milgrom J (2008). Risk factors for antenatal depression, postnatal depression and parenting stress. BMC Psychiatry.

[42] Austin MP, Hadzi-Pavlovic D, Leader L, Saint K, Parker G (2005). Maternal trait anxiety, depression and life event stress in pregnancy: relationships with infant temperament. Early Hum Dev.

[43] Loomans EM, van der Stelt O, van Eijsden M, Gemke RJ, Vrijkotte T, den Bergh BR (2011). Antenatal maternal anxiety is associated with problem behaviour at age five. Early Hum Dev.

[44] Talge NM, Neal C, Glover V, Early Stress TR, Prevention Science Network F, Neonatal Experience on C, Adolescent Mental H (2007). Antenatal maternal stress and long-term effects on child neurodevelopment: how and why?. J Child Psychol Psychiatry.

[45] O'Connor TG, Heron J, Golding J, Beveridge M, Glover V (2002). Maternal antenatal anxiety and children's behavioural/emotional problems at 4 years. Report from the Avon Longitudinal Study of Parents and Children. Br J Psychiatry.

[46] Clark J (2003). How to peer review a qualitative manuscript.

[47] National Health Call Centre Network (2010). The use of online health information and call centres in Australia: 18 November 2010.

[CR48] The pre-publication history for this paper can be accessed here:http://www.biomedcentral.com/1471-2393/14/318/prepub

